# Immunophenotyping of an Unusual Mixed-Type Extraskeletal Osteosarcoma in a Dog

**DOI:** 10.3390/vetsci8120307

**Published:** 2021-12-06

**Authors:** Hyo-Sung Kim, Han-Jun Kim, Hyun-Jeong Hwang, Jong-Hyun Ahn, Sun-Hee Do

**Affiliations:** 1 Department of Veterinary Clinical Pathology, College of Veterinary Medicine, Konkuk University, Gwangjin-gu, Seoul 05029, Korea; w6373515@naver.com (H.-S.K.); vet.hanjun@gmail.com (H.-J.K.); ibbng@naver.com (H.-J.H.); 2 Terasaki Institute for Biomedical Innovation, Los Angeles, CA 90064, USA; 3 Waltz Animal Hospital, Yeongdeungpo-gu, Seoul 07411, Korea; forcep1130@naver.com

**Keywords:** dog, extraskeletal, hypoxia, immunohistochemistry, osteosarcoma, prognosis

## Abstract

A 6-year-old female Maltese dog presented with a cervical mass without pain. The tumor was surrounded by a thick fibrous tissue and consisted of an osteoid matrix with osteoblasts and two distinct areas: a mesenchymal cell-rich lesion with numerous multinucleated giant cells and a chondroid matrix-rich lesion. The tumor cells exhibited heterogeneous protein expression, including a positive expression of vimentin, cytokeratin, RANKL, CRLR, SOX9, and collagen 2, and was diagnosed as extraskeletal osteosarcoma. Despite its malignancy, the dog showed no sign of recurrence or metastasis three months after the resection. Further analysis of the tumor cells revealed a high expression of proliferation- and metastasis-related biomarkers in the absence of angiogenesis-related biomarkers, suggesting that the lack of angiogenesis and the elevated tumor-associated fibrosis resulted in a hypoxic tumor microenvironment and prevented metastasis.

## 1. Introduction

Osteosarcomas are classified into multiple types and subtypes based on their histological patterns [1]. However, whether the histological type is associated with prognosis is not fully understood [2,3]. Researchers have attempted to identify independent diagnostic markers, such as ezrin [4], galectin-1 [5,6], osteonectin and osteocalcin [7], and p63 [8], and prognostic markers, such as COX-2 [9,10], ezrin [11,12,13], FGF-2 [14], IGF2 [15], RUNX2 [16], SOX9 [17], and VEGF [18]. Owing to the diverse and expansive immunoprofile of osteosarcoma, it is difficult to reach a consensus regarding the identification and selection of ideal markers for this disease [1,19,20,21]. Therefore, a more comprehensive study and further investigations are warranted so that we can detect and conclusively establish the role of these markers.

Extraskeletal osteosarcoma can be diagnosed based on the following criteria: no connection to the skeletal system, neoplastic bone/osteoid, and malignant cells [1]. Extraskeletal osteosarcoma in dogs is a highly malignant neoplasm, wherein the median survival time is only 26 days [22]. The tumor extensively infiltrates the surrounding tissue, making excision extremely difficult, thereby leading to local recurrence with euthanasia as the major cause of death.

Mixed osteosarcoma exhibits both histological characteristics and is generally diagnosed when it cannot be classified as something else [23]. In the present report, we describe an extremely rare type of extraskeletal osteosarcoma—a mixed subtype rich in chondrocytes and giant cells—in a Maltese dog. To the best of our knowledge, this type of osteosarcoma has not been reported in the veterinary literature, although a similar case was previously reported in human medicine [24]. Therefore, we characterized the expression of the diagnostic, prognostic, and therapeutic markers using immunohistochemistry. We also discuss the significance of the present report in the light of previous studies.

## 2. Case Presentation

**Figure 1:**
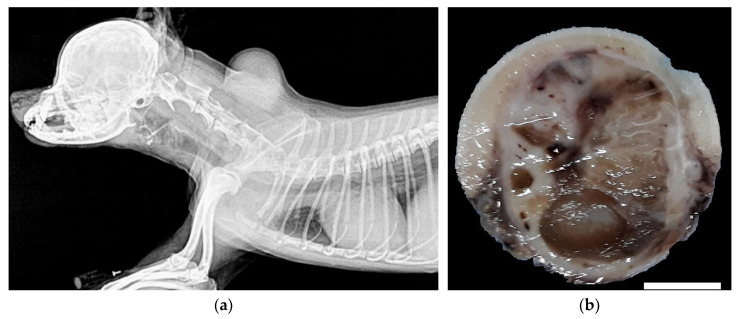
Subcutaneous tumor on the cervical region of a Maltese dog: (**a**) radiographic image of the lesion, revealing that the spherical tumor on the neck has no relation to other tissue, such as the cervical bone or muscle; (**b**) macroscopic appearance at the cut surface of the mass. The tumor consists of white and translucent tissue, with brown gelatinous material at the center. Scale bar = 10 mm.

A 6-year-old female Maltese dog presented with the primary complaint of a cervical mass without pain. The dog did not have a history of injection into the cervical area, trauma, or irradiation. A spherical mass right beneath the skin was identified using radiography, which showed soft tissue density with a few mineralized foci (Figure 1a). The subcutaneous mass was attached to the skin, but it was not adherent to other tissues, such as the cervical bone or muscle, and no evidence of tumor metastasis, invasion, or other abnormalities was observed. The serum chemistry showed no significant changes except for elevated alanine aminotransferase (225 U/L). The mass continued growing despite a two-week course of antibiotic (cephalosporin, 22 mg/kg, bid) and non-steroidal anti-inflammatory drug (prednisolone, 0.5 mg/kg, bid) treatment; therefore, surgical resection was performed. The tumor was easily separated from the underlying muscle, fixed in 10% neutral-buffered formalin, and transported to our laboratory for histopathological examination.

Upon gross examination, we found that a spherical tumor, 3 cm in diameter, was attached to the skin. The tumor consisted of rubbery to moderately firm tissue that was transparent or cream colored, with scattered focal hemorrhagic foci. The inside of the tumor was occupied by a dark-brown, gelatinous material (Figure 1b). The cross-sections that were taken from more than eight different positions of the tumor were processed routinely for paraffin embedding. Sections, 4 µm in thickness, were prepared and stained with hematoxylin and eosin.

**Figure 2:**
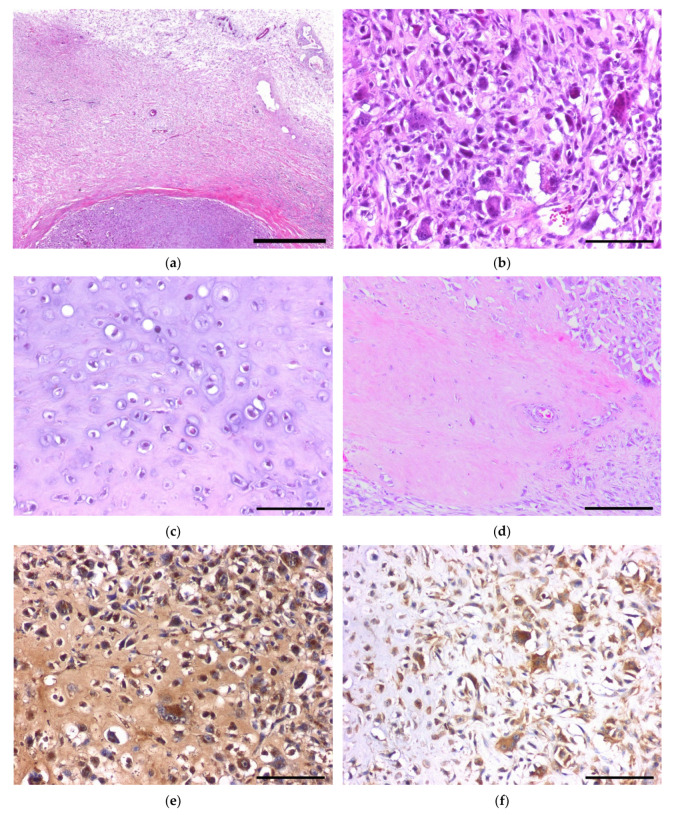
Histopathology of extraskeletal osteosarcoma and reference images of immunohistochemistry for biomarkers: (**a**) thick fibrous tissue surrounding the neoplasm; (**b**) hypercellular lesion with pleomorphic mesenchymal cells and multinucleated giant cells; (**c**) chondroid matrix-rich lesion in the deeper portion of the extraskeletal osteosarcoma; (**d**) small portion of the tumor-forming osteoid matrix with lacuna and osteoblast lining; (**e**) strong positive immunoreaction for RANKL on both tumor cells and stroma; (**f**) cytoplasmic positive reaction for ezrin. Scale bar = 1 mm (**a**), 100 µm (**b**,**c**,**e**,**f**), and 200 µm (**d**).

Analysis at the microscopic level showed that the tumor was located on subcutaneous fat tissue. It also showed that it was irregularly lobulated, well circumscribed and encapsulated by the thick fibrous tissue (Figure 2a). There was no adhesion or connection with surrounding tissues, such as muscle or cartilage. The lobules consisted of two distinct areas, namely, a mesenchymal cell-rich lesion and an extracellular matrix-rich lesion (Supplementary Materials, Figure S1a). The two regions were gradually admixed without an abrupt transition in cellularity or cell morphology. In the hypercellular portion of the tumor, many medium-sized fusiform to polyhedral mesenchymal cells were observed, along with numerous multinuclear giant cells (MGCs), which contained three to 20 nuclei (Figure 2b). The mesenchymal cells were randomly arranged and contained lacy chromatin, prominent nucleoli, and scant cytoplasm. The tumor cells showed marked atypia, including anisocytosis, anisokaryosis, and nuclear pleomorphism. A few atypical mitotic figures (Supplementary Materials, Figure S1b) with a mitotic activity index of 20/10 high-power field (0.237 mm^2^) were observed on the mesenchymal cell-rich lesion at the periphery [25]. The MGCs also had various subtypes that resembled Langhans, foreign body, and osteoclast types. In the matrix-rich lesion, the characteristic cells with lacunae-like clear to eosinophilic cytoplasm were scattered in an amorphous, lightly basophilic to eosinophilic extracellular matrix. The nuclei were pleomorphic, ranging from small lymphocyte-like nuclei to enlarged polyhedral types, and from hyperchromic to vesicular, indicating chondroid differentiation (Figure 2c and Supplementary Materials, Figure S1c). Chondrocytes were moderately polymorphic and varied in their size and chromatin pattern. Mitosis was minimal in the chondrocytes, whereas myxoid degeneration to liquefaction was observed at the deep portion of the chondroid matrix. In addition to the tumor’s two different principal components, a highly eosinophilic, dense matrix was observed (Figure 2d and Supplementary Materials, Figure S1d,e). This was considered to be an osteoid matrix, as it appeared that amorphous and partially calcified, osteoblast-like small cells were lined along the matrix, and lacuna-like chambers were present in the matrix. Other staining methods were used to confirm the two different matrices. The extracellular matrix in the hypocellular lesion was stained blue with PAS (Periodic acid-Schiff)-Alcian blue (pH 2.5, BBC Biochemical, Mt Vernon, WA, USA) (Supplementary Materials, Figure S1f) and Masson’s trichrome staining (BBC Biochemical) (Supplementary Materials, Figure S1g), showing the same staining properties as those of the chondroid matrix. The osteoid matrix exhibited a light-blue to pink color after PAS-Alcian blue staining (Supplementary Materials, Figure S1f), and a patchy red/blue color after Masson’s trichrome staining (Supplementary Materials, Figure S1g). After using von Kossa staining to reveal the calcium, a few calcified foci of the lesion were stained black (Supplementary Materials, Figure S1h).

To identify the tumor’s nature, we further analyzed the protein expression using immunohistochemistry. The detailed information on the primary antibody and antigen retrieval method is listed in the Supplementary Materials, Table S1. Briefly, sections were deparaffinized, rehydrated, and heated in a sodium citrate buffer (10 mM, pH 6.0) or tris-EDTA buffer (10 mM, pH 9.0) using a microwave (600 W), or were otherwise incubated at 37 °C in a humidified chamber with pepsin (0.5%) or proteinase K (25 µg/mL) to unmask the antigen. After antigen retrieval, the tissue sections were incubated in 3% hydrogen peroxide for 30 min to quench the endogenous peroxidases, blocked for 30 min using 2.5% normal serum, and incubated overnight at 4 °C with the primary antibody. The antibody-conjugated sections were then labeled using a VECTASTAIN^®^ ABC-HRP Kit (Vector Laboratories, Burlingame, CA, USA) following the manufacturer’s instruction. The signals were visualized with a DAB substrate (Vector Laboratories) and counterstained with Mayer’s hematoxylin (ScyTek Laboratories, Logan, UT, USA). The immunohistochemistry results are listed in Table 1 and the photomicrographs are shown in the Supplementary Materials, Figure S2. Neoplastic cells expressed both vimentin and pan-cytokeratin, along with mesenchymal and epithelial markers, respectively, which is consistent with previous reports which state that extraskeletal osteosarcoma can express different protein types depending on its subtype, origin, and site [26]. The receptor activator of nuclear factor kappa-B ligand (RANKL), one of the critical cytokine ligands that induces osteoclast differentiation [27,28], was highly expressed in most tumor cells (Figure 2e), along with a calcitonin receptor-like receptor (CRLR), a marker of osteoclasts that differentiates it from foreign body MGCs [29]. Moreover, SOX9 and collagen type 2, which are markers for chondrosarcoma, were highly expressed in the tumor cells, while S100 was mildly expressed. While α-SMA, a marker for identifying myoepithelioma, was mildly expressed, CD34, which is a marker for identifying epithelioid sarcoma, malignant solitary fibrous tumor, and Ewing sarcoma [30], was not detected (Table 1).

## 3. Discussion

The accurate diagnosis and classification of osteosarcoma are complicated due to morphological variations in the histological sections and the detection of unusual biomarkers [7,31]. We excluded mesenchymal chondrosarcoma due to the osteoid deposition and the lack of a hemangiopericytomatous pattern, which is a compact arrangement that surrounds the sinusoidal vessels [20,23,32,33]. In rare cases, a tumor with numerous giant cells could be mistaken for a giant-cell tumor, meaning that the malignant osteosarcoma cells might go unnoticed. Significantly, a giant-cell tumor that affects the tendon sheath can also exhibit the same markers as a chondroid matrix formation tumor [25,34]. However, we excluded the possibility of it being a giant-cell tumor because the tumor produced a considerable amount of extracellular matrices with true cartilaginous differentiation, while the MGCs showed marked pleomorphism [35]. We also ruled out other minor tumor types, such as sclerosing rhabdomyosarcoma, myositis ossificans, and ossifying fibromyxoid tumors, due to their unique histological patterns and origin [30]. There was no histological evidence of injection-site sarcoma, such as necrosis surrounded by macrophages, foreign materials observed using polarized microscopy, or a history of trauma and radiation according to the medical records [31]. The absence of a fibrosarcoma-like lesion was also characteristic, helping us differentiate it from the injection-site sarcoma that occurs in cats, or undifferentiated pleomorphic sarcoma [28,36]. Extraskeletal osteosarcoma can be diagnosed from sarcomatous proliferation with a high mitotic index and osteoid production without skeletal involvement [22,37]. Therefore, the tumor was finally diagnosed as mixed-type osteosarcoma (giant-cell rich and chondroblastic), primarily originating from the extraskeletal region.

Extraskeletal osteosarcoma is a highly malignant tumor with frequent metastasis [28,31,38]. However, the dog had no sign of recurrence nor metastasis after a routine X-ray examination performed three months post-surgical resection. Therefore, we investigated other prognostic biomarkers in the isolated cancer cells. Ki67, a marker of cell proliferation, is a prognostic factor, and when the percentage of cells expressing this marker exceeds 30%, the cancer is considered to be at grade 3 [39]. In our case, the Ki67 proliferation index was 32.5% when counting 1000 nuclei with the exclusion of cytoplasmic staining. Sustained exposure to FGF-2 enhances cancer cell proliferation, induces drug resistance in tumor cells, and reduces osteogenic differentiation [14]. However, in this case, this was associated with the small amount of osteoid production. RUNX2, ezrin, and SOX9 are valid prognostic markers for osteosarcoma that imply a higher risk of spread [16,17,20]. In this case, the tumor expressed high immunoreactivity to Ki67, FGF-2, RUNX2, ezrin, and SOX9, suggesting a high risk of proliferation, metastasis, resistance to therapy, and hence, poor prognosis (Figure 2f).

COX-2, which is involved in angiogenesis, invasion, metastasis, and resistance to apoptosis, was partially expressed in the tumor cells [25]. Notably, tumor size did not decrease after two weeks of prednisolone treatment, which was possibly due, or at least partially due, to the partial expression of COX-2. VEGF and IGF2 are also involved in the angiogenic process of the neoplasm [20,40]. Despite the expression of proliferative and metastatic factors, the neoplastic cells expressed COX-2 partially and did not express VEGF or IGF2. Another remarkable feature was the presence of dense fibrous tissue circumscribing the tumor. Tumor-associated fibrosis can be a physical barrier to the infiltration of lymphocytes [41]. Furthermore, tumors with extensive fibrosis are poorly vascularized, which induces hypoxia in the tumor microenvironment and suppresses the immune response [41]. In this case, chondrogenic differentiation and myxoid degeneration were found mainly at the tumor center, where hypoxia is maximized. It seems that hypoxic conditions had caused chondrogenic differentiation in the pluripotent tumor cells [42].

The tumor expressed protein markers that were related to proliferation and metastasis, with minimal expression of angiogenic markers. Although the lack of long-term follow-up constitutes an important limitation, invasion and metastasis were not identified in the imaging and histopathological analyses. This corroborates a previous finding that extraskeletal osteosarcoma in the dermal or subcutaneous region exhibits a better prognosis than others because total en bloc excision was possible [22]. These findings suggest that the tumor-associated fibrosis and lack of vasculature might have caused hypoxia in the tumor microenvironment and prevented metastasis, thereby contributing to a better prognosis [40,41,43].

The standard treatment for osteosarcoma includes neoadjuvant chemotherapy, surgery, and adjuvant chemotherapy [38]. Leucovorin rescue, doxorubicin, cisplatin, and ifosfamide are the primary options for osteosarcoma treatment [44], hence more specific approaches are warranted [45]. The paradigm of cancer therapy has evolved from non-selective chemotherapy to “precision and personalized medicine” based on molecular matching [46]. Molecular profiling helps to determine the optimal treatment and provides information regarding the behavior of tumors. However, its use is limited to certain types of cancer (e.g., colon, breast, and non-Hodgkin’s lymphoma) [46]. Therefore, other cancer types need to be investigated to help us better understand the complexity and heterogeneity of cancer. Osteosarcoma is one of the tumors for which additional subtypes and prognostic factors need to be identified [2]. However, even the appropriate diagnosis of osteosarcoma is complicated as the histological patterns of osteosarcoma may vary between tissue slides from different cut locations [7]. Moreover, osteosarcoma can express different protein types depending on its type, origin, and site.

## 4. Conclusions

Herein, we describe an extremely rare osteosarcoma that expresses both giant-cell-rich and chondroblastic morphology, and which originated from the extraskeletal region. Through immunohistochemical characterization, we observed that the tumor expressed various markers with a negligible expression of angiogenic markers. This study provides clues that angiogenetic factors may better predict prognosis than other factors, such as proliferation and metastasis, and that tumor-associated fibrosis may help prevent both angiogenesis and metastasis. Therefore, we propose that molecular typing, using antibody panels and accompanied with classical classification, may be used for prognosis prediction and the improvement of treatment strategies.

## Figures and Tables

**Table 1:** Immunohistochemical analysis of mixed-type osteosarcoma.

Protein	Marker	Potential Role	Mesenchymal Cells	MGCs	Chondrocyte
Vimentin	Diagnostic	Mesenchymal	+++	+++	+++
Pan-cytokeratin		Epithelial	+/−	+/−	++
RANKL		Osteogenic	++	+++	+++
CRLR		Osteogenic	+	++	+++
Collagen 2		Chondrogenic	+	+++	+++
S100		Chondrogenic	+	+	+
α-SMA		Myogenic	+/−	+/−	+/−
CD34		Angiogenic	−	−	−
Ki-67	Prognostic	Proliferation	++/−	+++/−	+/−
FGF-2		Proliferation	+++	+++	+++
RUNX2		Metastasis	+++	+++	+++
Ezrin		Metastasis	+++	+++	++
SOX9		Metastasis	+	+++	+++
COX-2		Metastasis Angiogenesis	+/−	+++/−	++
VEGF		Angiogenesis	−	−	−
IGF2		Angiogenesis	−	−	−

Immunohistochemical staining results were scored by intensity of individual cell type. +, pale staining; ++, moderate staining; +++, marked immunostaining; −, negative reaction; /, different immunoreaction of each cell even in the same type. MGCs: multinuclear giant cells.

## Data Availability

Not applicable.

## Supplementary Materials

The following are available online at https://www.mdpi.com/article/10.3390/vetsci8120307/s1, Figure S1: Histopathology of the extraskeletal osteosarcoma. Figure S2: Immunohistochemistry panel to elucidate the tumor’s nature. Table S1: Detailed immunohistochemistry methods.
