# Supplementary material for: Immunophenotyping of an Unusual Mixed-Type Extraskeletal Osteosarcoma in a Dog
Source: Vet Sci. 2021 Dec 6;8(12):307. doi: 10.3390/vetsci8120307 (PMC8707392; doi:10.3390/vetsci8120307)
Supplement: Supplementary file 1 [file vetsci-08-00307-s001.zip › vetsci-1478095-supplementary.pdf]

Supplementary Table S1. Detailed immunohistochemistry methods.

Protein	Company (Cat #, Clone)	Antigen retrieval	Antibody dilution
Vimentin	Dako (M0725, V9)	HIER (Sodium citrate)	1:100
Pan-cytokeratin	Abcam (ab86734, AE1/AE3 and 5D3)	HIER (Sodium citrate)	1:100
S100	Dako (IS504, polyclonal)	HIER (Tris/EDTA)	1:100
Collagen 2	Merk Millipore (AB746P, polyclonal)	PIER (Pepsin)	1:500
Sox9	Abcam (ab26414, polyclonal)	HIER (Tris/EDTA)	1:200
Ezrin	Invitrogen (MA5-13862, 3C12)	HIER (Sodium citrate)	1:250
CD34	Abcam (ab81289, EP373Y)	HIER (Tris/EDTA)	1:1000
IGF2	Santa Cruz biotechnology (sc-5622, H-103)	HIER (Tris/EDTA)	1:200
Ki-67	Abcam (ab15580, polyclonal)	HIER (Sodium citrate)	1:500
Runx2	Abcam (ab23981, polyclonal)	HIER (Sodium citrate)	1:200
VEGF	Santa Cruz biotechnology (sc-80439, VE01C)	HIER (Sodium citrate)	1:250
Cox-2	Santa Cruz biotechnology (sc-1747-R, polyclonal)	HIER (Sodium citrate)	1:50
FGF-2	Santa Cruz biotechnology (sc-79, polyclonal)	HIER (Sodium citrate)	1:500
CRLR	Novusbio (NLS6731, polyclonal)	HIER (Sodium citrate)	1:100
α-SMA	Sigma-Aldrich (CBL171, 1A4)	Without antigen retrieval step	1:500
RANKL	Santa Cruz biotechnology (sc-7628, polyclonal)	PIER (Proteinase K)	1:250

HIER, heat-induced epitope retrieval; PIER, proteolytic-induced epitope retrieval.

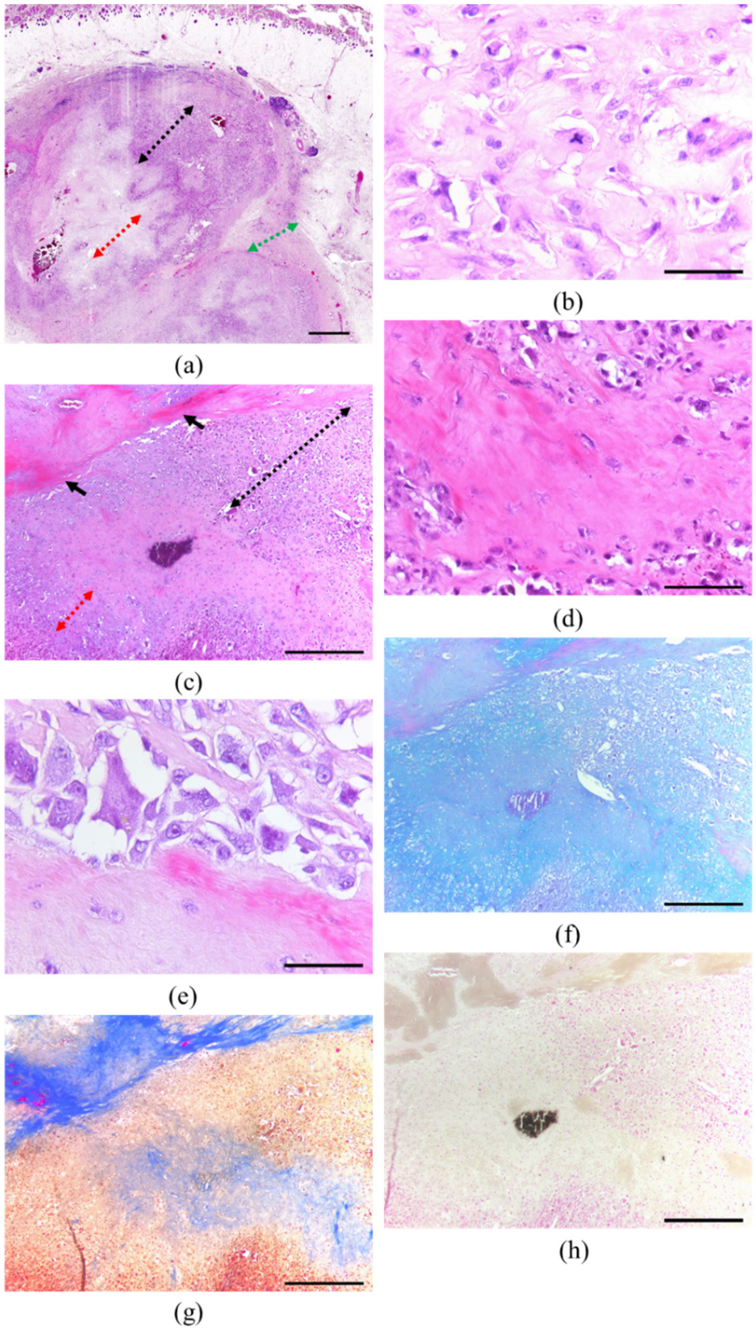

Supplementary Figure S1. Histopathology of the extraskeletal osteosarcoma. (**a**) The tumor was located on the subcutaneous adipose tissue surrounded by thick fibrous tissue (green dashed arrows). It was irregularly lobulated and composed of two distinct areas: hypercellular (black dashed arrows) and hypocellular (red dashed arrows) lesions; (**b**) Tripolar mitosis within the pleomorphic mesenchymal cells; (**c**) Highly eosinophilic, osteoid matrix (black arrow) is found along with giant cells (black dashed arrows) and chondrocytes (red dashed arrows); (**d**) Microscopic appearance of osteoid matrix; (**e**) Osteoblast-like cells along with osteoid matrix; (**f**) Chondroid tissue shows positive blue color via PAS-Alcian blue staining; (**g**) Osteoid matrix exhibits patchy red/blue color via Masson's trichrome staining; (**h**) Few mineralized foci in the osteoid are shown as black colored-calcium deposition in Von Kossa staining. Scale bar = 2 mm (**a**), 50 μm (**b**,**e**), 500 μm (**c**, **f**-**h**), and 100 μm (**d**).

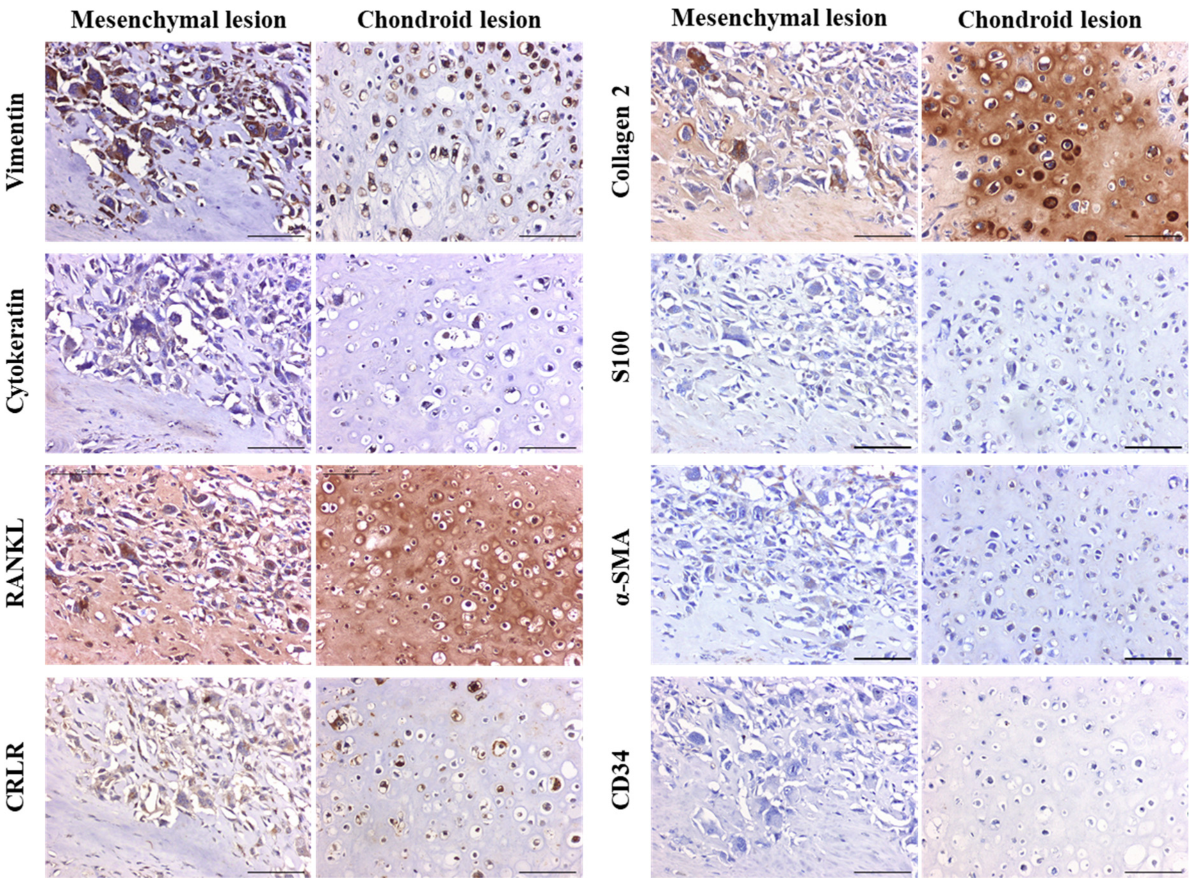

Supplementary Figure S2. Immunohistochemistry panel to elucidate the tumor's nature. The serially obtained tissue sections were labeled with diagnostic markers. The immunoreaction score is listed in Table 1. Scale bar = 100 μm.

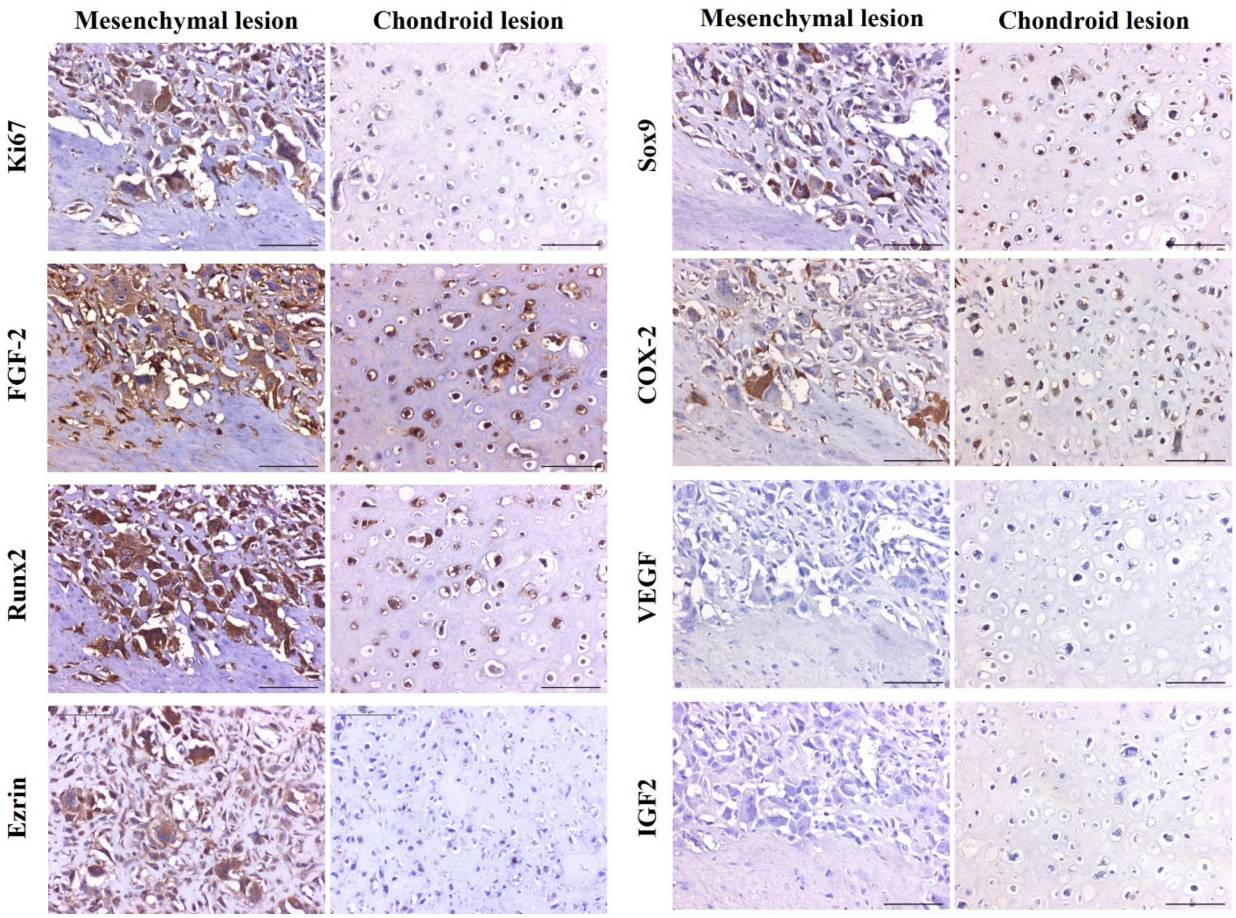

Supplementary Figure S2 (cont.). The serially obtained tissue sections were labeled with prognostic markers. Scale bar = 100 μm.
